# Artificial intelligence in heart failure

**DOI:** 10.1186/s43044-026-00723-w

**Published:** 2026-03-01

**Authors:** Xueqin Li, Yu Liu, Xianya Zhang, Na Yang, Tong Xu, Xinwu Cui, Gongquan Chen

**Affiliations:** 1 Department of Medical Ultrasound, Minda Hospital of Hubei Minzu University, Enshi, Hubei China; 2 Department of Medical Ultrasound, Tongji Hospital of Tongji Medical College, Huazhong University of Science and Technology, Wuhan, Hubei China; 3 Department of Ultrasound, Affiliated Hospital of Jilin Medical College, Jilin Jilin, China

**Keywords:** Heart failure, Artificial intelligence, Machine learning, Patient management

## Abstract

**Background:**

Heart failure (HF) affects millions of individuals worldwide and shows an increasing trend, constituting a serious public health issue. Considerable attention has been paid to the screening, diagnosis, risk prediction, treatment, and prognosis of HF. Although many guidelines for the management of HF have been proposed in recent years, the efficacy of evidence-based treatments seems to vary among patients. Therefore, the era of “one-size-fits-all” approaches is drawing to a close, and the concepts of precision medicine and individualized medicine are gradually taking root. Artificial intelligence (AI) is an emerging discipline in the rapidly growing field of computer science. It has now become deeply involved in all aspects of cardiovascular disease research, with particular relevance to HF, though its translation into clinical practice is yet to be fully realized. Although the use of AI in cardiovascular disease (CVD) and HF patient care, as well as cardiac resynchronization therapy (CRT), has been extensively discussed, a discussion from the standpoint of all aspects of HF clinical process is lacking.

**Main body:**

This review provides a comprehensive overview of the use of AI in HF in specific scenarios, including patient diagnosis, subtyping, prognostic assessment, pre- and post-treatment evaluation, and telecare. It also presents the prospects and challenges for the development of AI in the field of HF, with the expectation that a mature AI diagnosis and treatment system adapted to clinical practice will be developed in the future through in-depth research and validation.

**Conclusions:**

This review summarizes the application of AI in various links of HF management from diagnosis to telecare, and analyzes its current application limitations, existing challenges and future research directions, aiming to provide a reference for the subsequent clinical transformation and research optimization of AI in the HF field.

**Supplementary Information:**

The online version contains supplementary material available at 10.1186/s43044-026-00723-w.

## Background

Heart failure (HF) affects more than 37.7 million individuals worldwide and shows an increasing trend, constituting a serious public health issue [1–3]. For instance, the prevalence of HF among Chinese people aged ≥ 35 years has been reported to be 1.3% [4]. Considerable attention has been paid to the screening, diagnosis, risk prediction, treatment, and prognosis of HF. Although evidence-based treatment has improved the prognosis of HF patients, it has failed to stop the progression of the disease [5]. Advanced HF accounts for 1–10% of all HF patients [6]. Although patients with HF present similar clinical symptoms, the etiologies of the disease vary considerably. Ischemic heart disease accounts for approximately 40% of the etiologies of HF, but the prognosis is also related to factors such as the HF population, comorbidities and phenotypic heterogeneity [7]. Cardiovascular disease (CVD) remains the leading cause of death among HF patients, although it has shown a downward trend over time. Non-CVD deaths continue to increase, especially for HF with preserved ejection fraction (HFpEF) [7]. HFpEF, which has hitherto been overlooked but may become the main type of HF in the future, needs to be managed immediately.

**Fig. 1:**
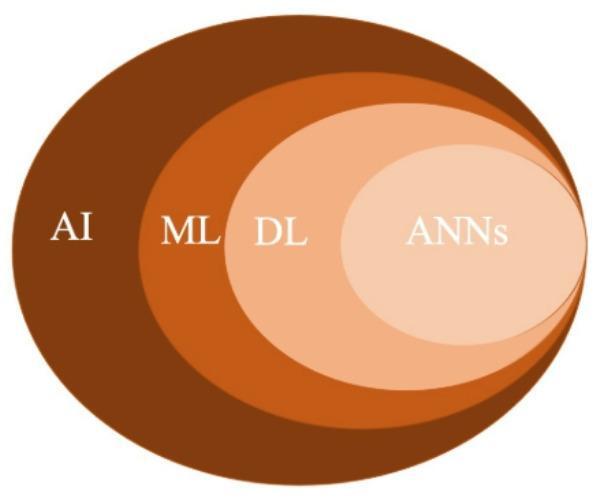
Inclusive relationships between *AI*, *ML*, *DL*, and *ANNs*. *AI* artificial intelligence, *ML* machine learning, *DL* deep learning, *ANNs* artificial neural networks

**Fig. 2:**
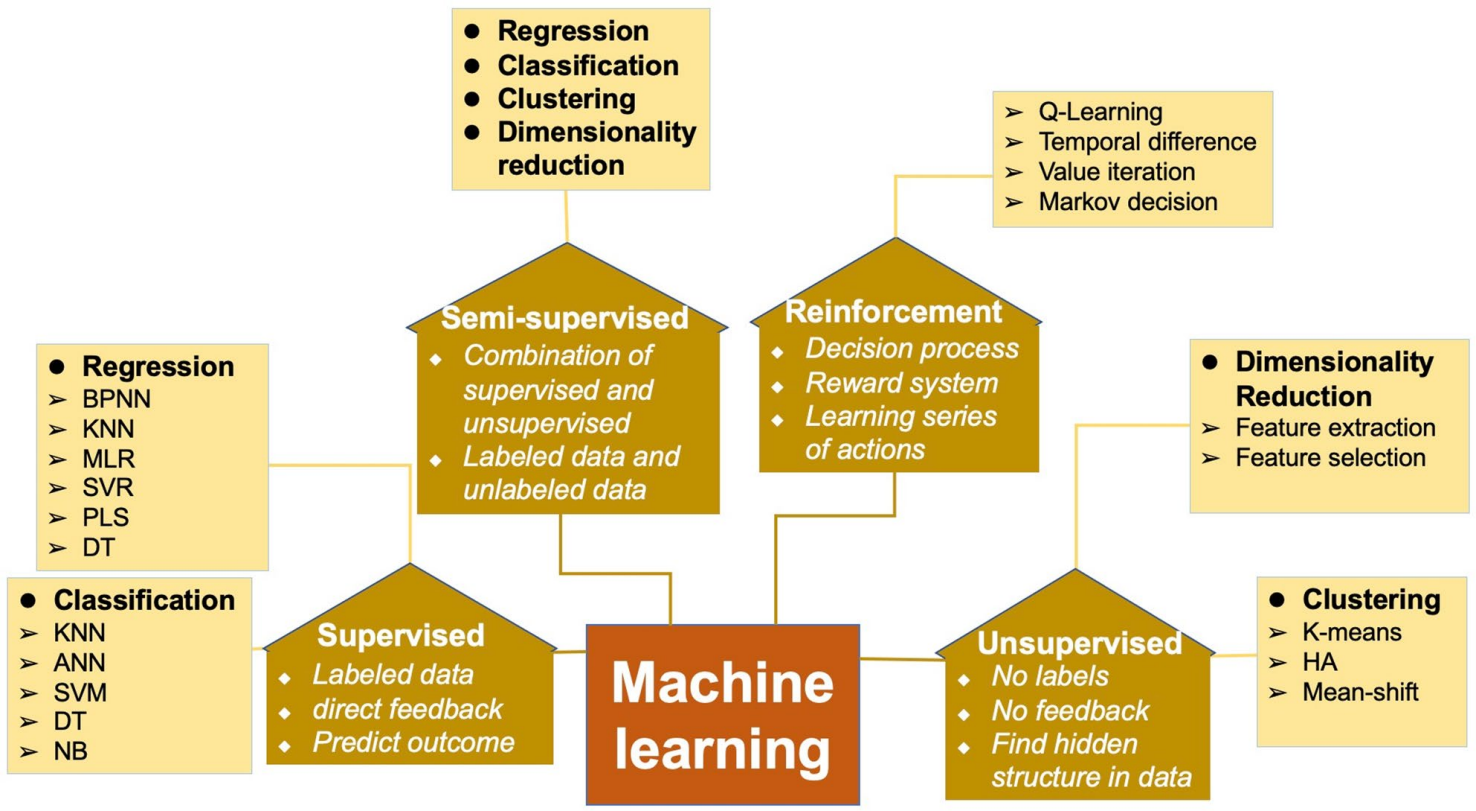
Types of machine learning ◆Characteristics of different types of machine learning ➢ Common algorithm ● The main tasks of machine learning *BPNN* back-propagation neural network, *KNN* k-nearest neighbor, *MLR* multiple linear regression, *SVR* support vector regression, *PLS* partial least squares, *DT* decision tree, *ANN* artificial neural network, *SVM* support vector machine, *NB* naive bayes, *HA* hierarchical algorithm

Although traditional statistical methods have made significant contributions to the understanding, risk assessment, and prognosis of HF, their ability to utilize and process data is limited. With the advent of big data, artificial intelligence (AI) has overcome the limitations of traditional statistical methods by utilizing large amounts of raw and unstructured data, mimicking human thinking and learning processes, and pooling knowledge [8].AI can reveal complex interrelationships and interactions between multiple variables through correlations between variables and specific outcomes [9, 10].It exhibits unique advantages in processing huge or complex datasets that are beyond humans’ processing capacity. Humans normally use one-to-one, one-to-many, or many-to-one models to explain the relationships between problems in steps, whereas AI can interpret the complex interrelationships between many problems using many-to-many models. Machine learning (ML) is a particularly popular subfield of AI (Fig. 1) based on algorithms that analyze data and their attributes by mimicking the ability of humans to recognize patterns [11]. Four commonly used methods for solving different tasks are supervised, unsupervised, semi-supervised, and reinforcement learning [12]. Figure 2 shows the four types of ML and their characteristics.

**Fig. 3:**
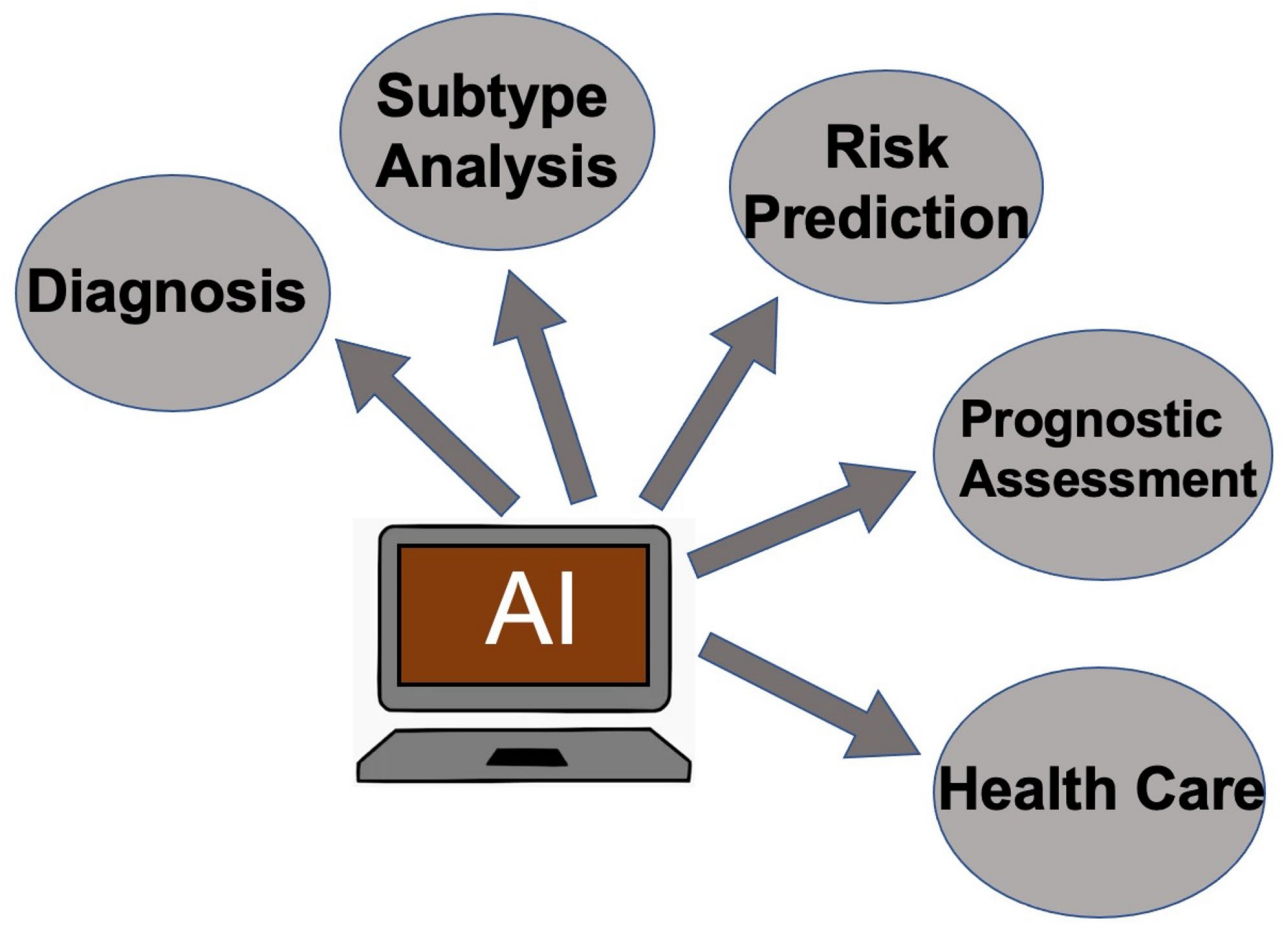
The use of AI in HF

Advancements in science and technology have promoted the increasing use of AI in the medical field, especially concerning CVD. It has mainly been used in cardiovascular imaging, arrhythmia detection, phenotypic analysis, and HF outcomes prediction [13, 14]. Although the use of AI in CVD and HF patient care, as well as cardiac resynchronization therapy (CRT), has been extensively discussed, a discussion from the standpoint of all aspects of HF clinical process is lacking. Therefore, this paper provides an overview of the application of AI in various links of HF management from diagnosis to remote care (Fig. 3), and summarizes the current application limitations, challenges and future research opportunities.

## Use of AI for heart failure diagnosis (screening)

HF is a complex syndrome that results from a structural or functional heart disease when appropriate treatment is not provided. HF is divided into HFpEF, HF with mid-range ejection fraction (HFmrEF), and HF with reduced ejection fraction (HFrEF) [15]. Relying on a single test or clinical feature to definitively diagnose HF may be inadequate. As science and technology continue to evolve, physicians expect to diagnose diseases more quickly, accurately, and efficiently. In this respect, AI shows potential application value for cardiologists in specific diagnostic scenarios [16].

The detection of HFpEF remains challenging. Although the H2FpEF score developed by Reddy et al. has shown favorable performance (better than the expert consensus-based algorithm) [17], it still fails to meet the needs of cardiologists. Previous studies have used conventional echocardiographic data [18], exercise stress echocardiography data [19, 20], plasma biomarker data [21]and heart sound characteristics [22]to identify HFpEF patients among seemingly healthy populations. Their findings suggest that these methods are difficult to raise the diagnostic performance to the desired level, even when combined with ML approaches. The AI model based on echocardiography (EchoGo Heart Failure v2) proposed by Ashley et al. exhibits better diagnostic performance than existing clinical scoring systems, including H2FPEF and HFA‑PEFF in the validated cohort [23]. Patients with a positive diagnosis by this model have a twofold increased risk of experiencing the composite endpoint events. Meanwhile, some scholars have also conducted in-depth research on electrocardiography (ECG). Unlike HFrEF, HFpEF often lacks distinct ECG signatures, making traditional diagnosis difficult. In 2021, the first deep learning convolutional neural network(DL-CNN) was developed to detect HFpEF (per ESC criteria) using standard 12-lead ECGs [24]. In external validation, the model showed high sensitivity (0.99) and negative predictive value (0.98), confirming its superior performance in HFpEF negative screening for specific study cohorts. An AI-ECG model, developed and validated to accurately identify left ventricular diastolic dysfunction grading and elevated filling pressure—including in HFpEF patients—exhibits prognostic value equivalent to echocardiography [25]. It effectively stratifies mortality risk in patients with inconclusive echocardiographic assessments and, with further optimization and validation, is expected to become a simple, promising adjunct diagnostic tool for cardiovascular diseases. Hong et al. further validated the value of 12-lead ECG-based AI models for HFpEF prediction in a large single-center cohort, with the model achieving an AUROC of 0.81 for HFpEF diagnosis and consistent performance across subgroups stratified by classic HFpEF risk factors; more importantly, the model could effectively stratify patients’ prognosis, with positive AI-ECG classified patients showing significantly higher 5-year risks of cardiac death and heart failure hospitalization [26].Gao et al. conducted a single-center study on a DL model based on a convolutional neural network-long short-term memory (CNN-LSTM) architecture. This model enabled the prediction of HFpEF risk via the input of 12-lead ECG signals [27]. The primary limitation to these investigations is that these AI models were predominantly constructed using highly selective, rigorously filtered cohort datasets, with inadequate external validation across diverse, unselected real-world populations. In addition, the models remain hindered by limited interpretability and poor clinical explainability. Therefore, further multicenter, large-sample, prospective studies are warranted to optimize the models’ tolerance to low-quality data and enhance their interpretability.

For the diagnosis of HFrEF, cardiologists have traditionally been more convinced of the value of echocardiography. Echocardiography remains the predominant examination for diagnosing HFrEF. However, the assessment of cardiac function based on echocardiography is physician-dependent and time-consuming. Tromp et al. developed a DL algorithm to automatically process dynamic ultrasound videos and spectral Doppler signals, but its diagnostic performance was not further improved compared with traditional methods [28]. Even more intriguing is the use of ECG. Attia et al. hypothesized that metabolic and structural disorders were associated with cardiomyopathic processes and might cause ECG changes that could be reliably detected by trained neural networks [29]. Driven by this assumption, an AI-enabled ECG data platform was created and prospectively validated [29, 30]. AI-enabled ECGs have also been used to identify Graves’ disease-related HFrEF, achieving a C-statistic of 0.85 in the study cohort [31]. Subsequent clinical trials have demonstrated the potential value of AI-enabled ECGs for the diagnosis of HFrEF in specific clinical scenarios [32, 33]. Hence, their application to HFrEF screening and emergency patients may achieve positive results after further validation. Combining single-lead electrocardiography embedded in a stethoscope with robust AI algorithms [33]is expected to become an essential tool for future HF screening. In addition, a recent study evaluated the use of AI applied to images of ECGs to predict HF risk, and the result showed that the higher model output probabilities were progressively associated with a higher risk for HF in the study population [34].

Moreover, Choi et al. built a hybrid (expert- and ML-driven) clinical decision system that showed better performance in distinguishing between HFpEF, HFmrEF, and HFrEF than separate expert-driven and ML-driven systems in the research cohort [35]. Zerina et al. identified congestive heart failure (CHF) patients in a general population using a random forest (RF) approach that achieved 100% classification accuracy in the selected sample [36]. Jarrel et al. used generative learning to create generative visual rationales to display the disease characteristics of CHF and visualize the distinction between disease and disease-free states [37]. Ren et al. constructed a balanced RF model for the early prediction of HF in acute coronary syndrome that showed a better performance than a linear logistic regression model in the study cohort [38]. Lovedeep et al. used AI to predict HF risk from single-lead electrocardiographic signals, and the AI model achieved incremental discrimination and improved reclassification for HF over the pooled cohort equations to prevent HF (PCP-HF) in the research population [39].

Based on the aforementioned evidence, AI outperforms traditional statistical methods and expert clinical judgment in in some HF diagnostic scenarios, delivering superior accuracy and efficiency through the integration of multidimensional clinical data (i.e., medical history, physical examination findings, laboratory indices, ECG, and echocardiography), but its performance is limited by research design and population selection. AI models exhibit the potential for early identification of HF in specific populations, which may enable clinicians to initiate timely interventional strategies, thereby helping to slow disease progression, reduce associated medical costs and ease the disease burden on patients. However, the clinical application of AI in HF diagnosis is hindered by its inherent limitation of poor interpretability and insufficient external validation. The integration of AI-driven mechanistic insights into the pathogenesis of distinct HF subtypes into AI-based HF diagnostic models may enhance clinicians’ confidence in the clinical utility of AI algorithms in cardiology practice. Li et al. focused on oxidative stress (OS), a pivotal pathological mechanism in HF, and integrated single-cell RNA sequencing and bulk RNA sequencing data with ML algorithms to clearly elucidate the mechanistic cascade of “OS-related genes - cell-specific expression - HF pathological progression” [40]. In another study targeting HFpEF, Hahn et al. adopted a metabolic perspective and identified impaired fuel metabolic flexibility as a core pathological mechanism in the myocardium of HFpEF patients through myocardial metabolomic analysis [41]. Furthermore, they validated the dysregulated expression of metabolism-associated genes by correlating metabolomic data with gene expression profiles, which provides critical mechanistic evidence for elucidating the pathophysiological basis of HFpEF. Future research should further capitalize on the advantages of AI in processing high-dimensional data (e.g., multi-omics datasets integrating genomics, transcriptomics and metabolomics) to investigate the molecular mechanisms underlying the association between key biomarkers and metabolic dysregulation, clarify the roles of cell-specific effects and intercellular communication pathways in HF progression, and ultimately bridge the gap between clinical diagnosis and mechanistic understanding of HF. This will facilitate the development of more precise and targeted therapeutic strategies for HF.

## Subtype analysis

In the case of CVD, ML has mainly been used for diagnosis and decision-making. Phenotypic studies have also received increasing attention. Shah et al. first demonstrated the utility of phenotype-mapping algorithms for HFpEF classification in specific research cohorts [42]. Many studies have used ML algorithms to group HFpEF patients according to various characteristics (see supplement Table 1) and to analyze different patient groups’ treatment responses and prognoses [43–49], providing a basis for subsequent subtype-specific research. Using unsupervised ML combined with protein biomarkers of HFpEF, Woolley et al. divided HFpEF into four subgroups [50]. Significant differences in clinical features and outcomes between the four subgroups revealed pathophysiological differences. Patients in subgroup 1 had diabetic nephropathy and a biomarker profile linked to certain inflammatory responses. Patients in subgroup 4 had a biomarker profile associated with increased PI3K/AKT pathway activity and the highest risk of hospitalization and death. This investigation provided insights into the partial pathophysiological basis of HFpEF and a research direction for subsequent specific treatment exploration. Given the heterogeneity of HFpEF, clinical trials are needed to verify whether ML algorithms can identify the clinical phenotype of HFpEF and guide individualized treatment. The discovery of the underlying mechanisms and more rational treatments, along with the development of a comprehensive classification system in collaboration with cardiologists, is expected to lay a foundation for the realization of individualized treatments for HFpEF.

## Risk prediction

The high readmission and mortality rates of HF patients place exceptionally heavy burdens on patients and the healthcare systems. Therefore, it is crucial to identify high-risk individuals early and administer appropriate therapies. Supplement Table 2 shows the application of AI in HF risk prediction in different research scenarios. For instance, studies have reported that 30-day readmission predictions may provide better identification of negative cases, while 180-day readmission predictions may provide better identification of positive cases [51]. In a previous study involving 10,757 individuals with HF, the rate of 30-day readmissions and deaths was 23.7% [52]. The research team attempted to develop a ML algorithm using eight variables to match the performance of previous models, yet failed to analyze 30-day readmission and mortality rates separately. With the continuous updating of ML algorithms, the performance of HF risk prediction models has been improved in specific research scenarios, with C-statistics ranging from 0.628 to 0.89 in different studies (the high value of 0.89 is only from a study based on echocardiographic indicators) [51–56]. However, the development of more robust prediction models with better performance is necessary. Despite this progress, the development of more robust and high-performance prediction models applicable to real-world clinical scenarios remains imperative. Against this backdrop, Lovedeep et al. developed an AI-ECG model to assess the risk of heart failure based on single-lead ECG data from multinational cohorts. The model features simple data requirements, and shows potential for application in developing countries and resource-poor regions after further on-site verification and optimization [57].

The aforementioned studies have predicted short-term risks for HF patients in specific research cohorts. Other studies have investigated 1-year and 5-years all-cause or cardiac-cause mortality using ML approaches [49, 58–63]. The variables used in these studies included the electronic medical records, ECGs, echocardiograms, and serological tests. These studies offer new research ideas for clinical decision-making and prognostic assessment. Leveraging ML algorithms to automatically read data from sources such as electronic health records (EHR) and cardiac images, and then combine them with genomics, proteomics, clinical risk factors, and wearable devices data is expected to increase risk prediction accuracy in clinical practice [64].

## Prognostic assessment

More research needs to be conducted to change the “one-size-fits-all” paradigm and to achieve precise treatment. No country’s guidelines for the treatment of HF are sufficiently detailed, probably because no big data study has provided conclusive evidence [65]. Various questions remain in clinical practice: How should different comorbidities be managed? How should combination therapy doses be administered to obtain the best results? Many attempts have been made to achieve precision therapy for HF. It is expected that medical-industrial integration will be more helpful. ML applications for assessing the prognoses of patients receiving pharmacotherapy, CRT, and heart transplantation (HT) in specific research scenarios are discussed below (see supplement Table 3).

### Prognostic evaluation of pharmacotherapy

Medication remains the basic treatment for HF patients. Most HFrEF patients respond well to drug therapy. However, those who fail to respond exhibit rapid and unpredictable deterioration. The high risks involved in early surgical treatment limit its application. However, effectively screening patients who do not respond to drug therapy and performing timely invasive surgical interventions may be crucial.

McGilvray et al. developed a DL model based on EHR variables to assist physicians in identifying HF patients who did not respond to pharmacotherapy, with the model achieving a C-statistic of 0.91 in the research cohort [66]. However, it was based on data from previous studies and did not assess the efficacy of different drugs for HF with different pathophysiological causes.For HFrEF, experts recommend initiating “quadruple therapy” as soon as possible. Previous studies have shown that β-blockers reduce mortality rates in HFrEF patients with a normal sinus rhythm but not in patients with atrial fibrillation (AF) [67], suggesting that AF is associated with an unfavorable prognosis [68]. Karwath et al. screened nine double-blind randomized controlled clinical trials of β-blockers and grouped the β-blocker responses of patients with and without AF using variational self-encoders and hierarchical clustering. This approach was shown to be effective in predicting β-blocker responses in HFrEF in the selected trial population [69]. Notably, AI-driven therapeutic optimization has been investigated as a strategy to address diuretic resistance, a common clinical challenge affecting over one-third of HF patients. Gelman et al. enrolled 10 HF patients (covering HFrEF, HFmrEF, and HFpEF subtypes) with diuretic resistance, using the second-generation AI app Altus Care™ to deliver personalized furosemide regimens with randomized dosages and administration times within pre-defined ranges [70]. The study’s core strength lies in its targeted focus on diuretic resistance, leveraging AI to optimize pharmacotherapeutic details, covering all HF subtypes, and adopting a user-friendly app-based intervention. As a proof-of-concept study with inherent limitations—including a small sample size, single-center design without a control group, and short follow-up—it only demonstrates the feasibility of the regimen, with definitive clinical efficacy requiring validation in large-scale randomized controlled trials. Other studies have investigated responses to various pharmacotherapies and the associated prognoses based on different subtypes of HFpEF in specific research cohorts (see supplement Table 3) [43, 47, 71].

Incorporating disease phenotyping and pharmacotherapy responses into a DL model to rapidly predict treatment outcomes in specific scenarios can help improve drug treatment responses and reduce unnecessary invasive surgical interventions after further clinical verification. Furthermore, using ML to assess EHR variables combined with physiological indicators predictive of advanced HF may provide more accurate invasive treatment options for patients who do not respond to medical therapy at an early stage in specific clinical scenarios.

### Cardiac resynchronization therapy (CRT)

Several clinical trials have demonstrated that CRT reduces HF-related morbidity and mortality [72, 73]. However, the CRT nonresponse rate is as high as 30% [74, 75]. The effectiveness of CRT and postoperative complications constitute a considerable concern for cardiologists.

#### Screening CRT candidates

The principle of maximum patient benefit is the foundation of all treatment strategies. Therefore, the assessment of CRT candidates is a critical step in clinical decision-making. Chao et al. built an intelligent classifier to distinguish between CRT responders and nonresponders based on speckle-tracking echocardiography in the research cohort [76]. Many studies have also attempted to predict the efficacy of CRT when screening candidates [77–82]. Compared with the 2013 guidelines [83], ML showed higher prediction accuracy and higher event-free survival discrimination in the specific study cohort [77]. Moreover, a comparison between CRT defibrillators (CRT-D) and implantable cardioverters-defibrillators (ICD) in terms of the risk of death or HF events showed that CRT-Ds were more effective in the primary outcome in the research population [78]. Furthermore, an evaluation of CRT candidates’ systemic right ventricular function showed that tricuspid annular plane systolic excursion was the main prognostic indicator [81], suggesting that patients should systematically undergo echocardiography to assess right ventricular size and function before receiving a CRT implant. The same study also assessed the limitations of previous clinical implementation protocols and answered some of the questions that exist in clinical practice. For instance, what examinations should be performed before CRT? What should be the focus of these examinations? Which device should be used: a CRT-D or an ICD? Certainly, these AI models need to be further optimized and validated in large-scale clinical populations before they are directly applied to clinical practice.

#### Postoperative outcome prediction and early identification of adverse outcomes

Cardiologists must keep track of patient’s clinical status following CRT. The early detection of indicators of adverse outcomes is of critical importance. The MultiSENSE study was an international, non-randomized prospective study conducted to evaluate the ability of CRT-D’s multisensors to detect heart status deterioration early [84]. The study assessed 100 HF events in 990 cases from 120 centers around the world to develop and validate a prediction algorithm, and 900 patients (development + test cohorts) were followed up for 1 year to build a predictive model. The model showed 70% sensitivity.The unexplained alarm rate was 1.47 alarms per patient year, and the median time until HF was 34 days [85]. HeartLogic’s multi-sensor index and alarm algorithm shows potential for the early prediction of heart decompensation after CRT implantation, and further research is needed to confirm the benefits of this algorithm for patients with HF receiving CRT implants.

At present, none of the ML methods used to predict CRT outcomes can meet clinical requirements (ROCs between 0.69 and 0.80) (see supplement Table 3) [82, 86–88]. With the data including demographics, medical history, physical condition, therapeutic medications, echocardiogram, and laboratory parameters not used, Wouters et al. used only standard 12-lead ECGs (ignoring demographic data, medical histories, physical conditions, medications, echocardiograms, and laboratory parameters) to assess the combined clinical endpoints of death, left ventricular assist devices, and HT after CRT [82]. Although the model’s predictive performance was imperfect (C-statistic 0.69), its metrics are simple and easy to manipulate, and it may become a otential tool for CRT outcome prediction in the future after improving diagnostic performance and integrating multi-dimensional data. The Multicenter Automatic Defibrillator Implantation Trial for CRT (MADIT-CRT) in the USA identified four HF patient subgroups with significant differences in clinical characteristics, biomarkers, cardiac structure and function, and clinical outcomes [78]. This study provided significant insights into whether CRT implantation should be performed and what outcomes should be expected after implantation in specific patient subgroups. After predicting the efficacy of CRT, Feeny et al. used ML to analyze 12-lead QRS waveforms and to identify CRT outcomes based on the limitations of the assessment for patient selection for QRS interval prolongation and left bundle branch block (LBBB) [89]. The early identification of HF events after CRT by ML in specific research scenarios provided a theoretical basis for timely clinical decision-making.

## Heart transplantation

Heart transplantation (HT) is the most effective treatment for patients with terminal HF. However, it is severely limited by various factors, such as a shortage of donor organs and low donor-recipient matching rates. To ensure the maximum utilization of medical resources, hospitalization length and survival rates after transplantation have become major concerns among cardiologists. Studies have attempted to predict the length of hospitalization and the risk of death after HT using ML in specific research cohorts [90–92]. Agasthi et al. showed that the length of hospital stay was the most crucial risk factor for death [93]. To assess the prediction of survival following HT, Medved et al. compared the International Heart Transplantation Survival Algorithm with the Index for Mortality Prediction After Cardiac Transplantation [94]. The International Heart Transplantation Survival Algorithm with the Index showed superior predictive performance in predicting 1-year survival and mortality after HT in the research cohort. Several other ML and DL models have achieved moderate 1-year mortality prediction performance with ROCs ranging from 0.63 to 0.77 [92, 95, 96]. The prediction of long-term (5-year) survival is also of interest to physicians and patients [93, 95]. A systematic review assessed the value of AI in predicting HT failure and mortality and guiding posttransplant treatment [97]. It has been reported that estimating the benefits of HT based on preoperative laboratory results and imaging data using AI can boost patient confidence and subsequent treatment compliance [97]. However, the use of AI in predicting HT outcomes in patients with advanced HF remains limited because few patients undergo HT each year, whereas AI algorithms rely on big data. Cooperation between multiple HT centers can significantly contribute to developing robust models. Effective AI models optimized through multi-center data integration are expected to improve the accuracy of clinical decision-making and the efficient allocation and management of healthcare resources for advanced HF.

## Heart failure care

Researchers at NewYork-Presbyterian have been working to employ AI to streamline clinical operations in cardiac care based on real-time data-driven prediction strategies, which can increase access to care by reducing the bottlenecks in care that occur in clinical settings [98]. The Patient Self-care uSing eHealth In chrONic Heart Failure (PASSION-HF) aimed to develop a virtual family doctor system focused on prediction, prevention, and personalized therapy based on HF management guidelines and effective HF patient self-care [99]. A rigorous classification of HF based on the patients’ histories was planned to provide individualized treatment and self-care measures, and the potential of this patient-centered management model to improve patient engagement and well-being needs to be verified by subsequent clinical research.

TRIAGE-HF was the first study to prospectively analyze a correlation between heart failure risk status (HFRS) related to implantable electronic devices and clinician-assessed HF decompensation [100]. HFRS provided a convenient management model for patients in the study cohort and served as a theoretical basis for remote monitoring for the early detection of heart status deterioration in clinical practice.

Meanwhile, wearable technologies for the management of HF are expected to attract increasing reseach interest [101]. Recently, the University of California developed a wearable cardiac ultrasound device for continuous, real-time, and direct cardiac function assessment [102]. This device enabled the acquisition of uninterrupted frame-by-frame cardiac images of patients in various environments and made it more convenient to monitor cardiac function without the need to rely on bulky cardiac ultrasound equipment. This noninvasive device also shows application potential for outpatients and individuals engaging in exercise. after further clinical validation Wearable sensors can provide copious amounts of clinical data (heart rate, blood pressure, sleep quality, etc.) for HF management and provide additional information for epidemiological studies. As material science and computer technology advance rapidly, data derived from wearable devices, when integrated with AI algorithms, may yield valuable research insights for the management of HF after large-scale clinical validation and practical application testing.

## Current challenges and future trends

AI has shown certain application value for the diagnosis, classification, risk assessment, and prognosis of HF patients in specific research scenarios. However, most models suffer from a lack of generalization and limited clinical interpretability, and AI has not yet been fully integrated into clinical practice for the management of HF. Applying AI to develop precise and individualized treatment protocols for HF remains a major challenge that requires in-depth research and multi-center validation. To create HF management models that are as intelligent and practical as face recognition systems, more robust and diverse clinical data support is required. Based on the learning behavior characteristics of reinforcement learning trial and error, this work may explore the potential application of reinforcement learning algorithms, and the current application of such algorithms in medicine is relatively limited, requiring basic research and clinical transformation to verify their feasibility. It is possible that making clinical decisions based on the characteristics of HF patients could be a promising research direction of reinforcement learning algorithms, particularly for dynamically optimizing long-term treatment strategies.Another significant issue is that biases inherent in the data may affect the model-building process, leading to inaccurate results and potentially exacerbating healthcare disparities. Therefore, cardiologists need a deeper understanding of the mechanisms of HF and keen clinical insight to identify errors in the AI training process. Furthermore, closer collaboration between clinicians and AI developers is essential to design algorithms that incorporate clinical knowledge. Extensive evaluation and validation, including rigorous multicenter prospective studies in real-world settings, are necessary before integrating AI into HF clinical practice, which place additional burdens on developers. Finally, ethical considerations cannot be overlooked. UNESCO’s *Recommendation on the Ethics of Artificial Intelligence* enshrines the right to privacy and data protection, human oversight and determination, and transparency and explainability as its core principles [103]. All clinical data used for model training shall be strictly desensitized and managed in compliance with data security laws, which constitutes the fundamental ethical bottom line for the application of AI in HF management. As an auxiliary tool, AI must not replace humans in making core clinical decisions; its role should be clearly defined as a decision-support system for “augmented clinical intelligence”.

**Fig. 4:**
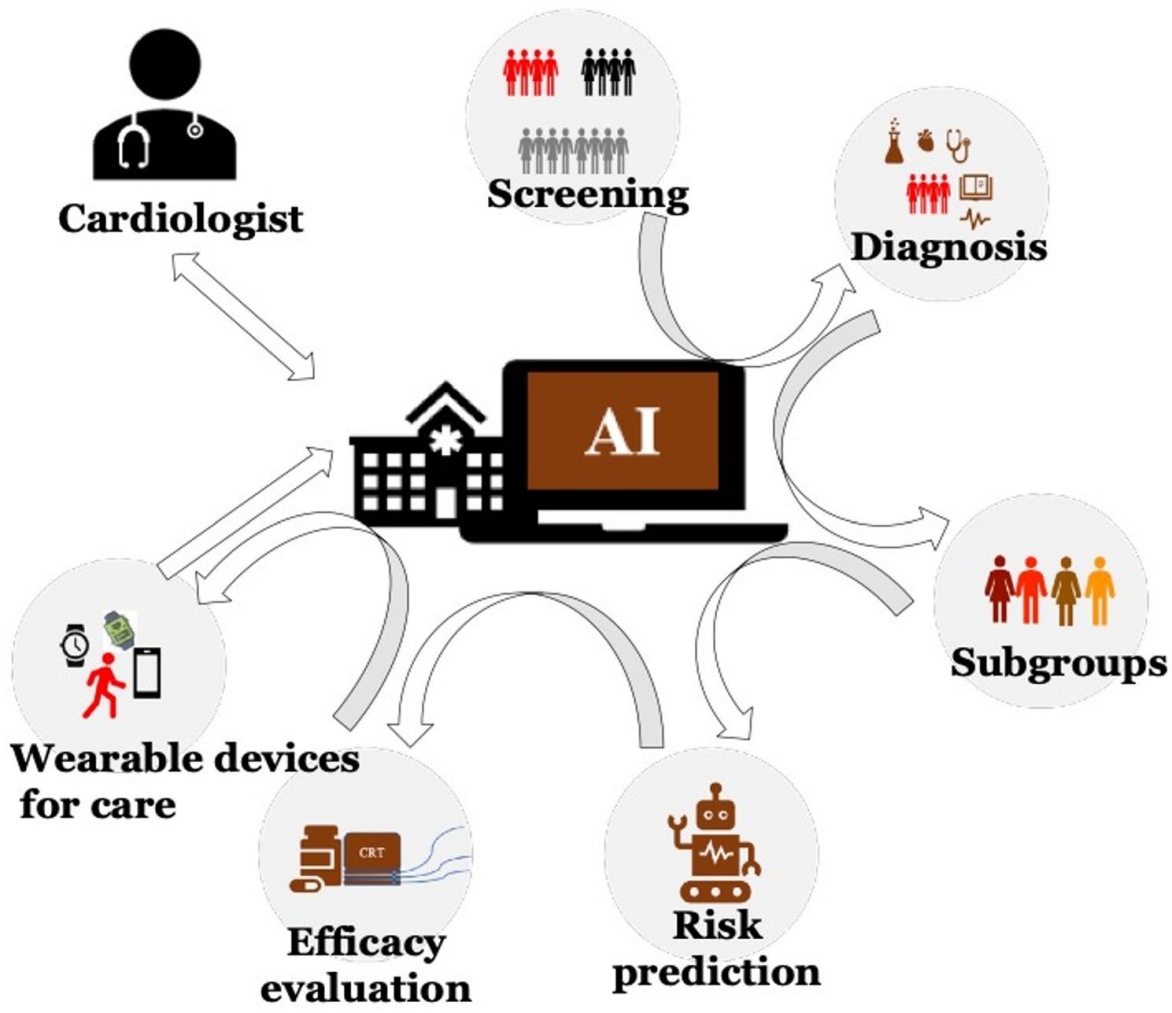
Application and workflow of AI in HF patients from screening to care. At each step from HF screening to telecare, the AI system feeds information to a cardiologist who gives instructions. The AI then performs the next step of pre-qualification until the patient transmits the information via a wearable device for remote care

Although AI in HF clinical practice faces certain challenges, it also offers numerous opportunities. First, as HER systems become more standardized and interoperable, and with the emergence of new data sources like wearable devices, structured and unstructured data are becoming more accessible. Thus, AI is expected to become a crucial component of the HF diagnostic and treatment workflows after sufficient clinical validation and model optimization. This will allow cardiologists to devote more time to patient management and personalized consultation, thereby improving the physician-patient relationship and treatment adherence. Second, AI can address complicated diagnostic and therapeutic problems that are excessively challenging for humans in specific scenarios, such as identifying subtle prognostic patterns from multimodal data. By outperforming the human brain in processing speed and analytical capability, AI can assist in predicting outcomes and enabling earlier interventions for HF patients. Finally, as shown in Fig. 4, when integrated into future clinical practice as an embedded backend engine or a clinical decision-support system after overcoming current technical bottlenecks, AI can play an important auxiliary role in the precise treatment and personalized management of HF. A systematic therapy regimen can be established akin to a clinical pathway, where AI provides actionable recommendations at each step in specific clinical scenarios, and the physician decides whether to adopt them based on the patient’s overall clinical context.

## Limitation

This review summarizes the applications of AI in HF management, yet it has inherent limitations, together with the notable constraints of the AI-based HF research field itself. First, this review has methodological biases: included literature is mainly English-language, potentially omitting evidence from non-English regions; no stratified analysis by the methodological quality and evidence level of original studies was performed, and rapid advances in AI may have left some newly emerging evidence uncovered. Second, AI models for HF are mostly developed on highly selected single-center cohorts with insufficient real-world external validation, leading to poor generalizability. Most are “black-box” algorithms with limited clinical interpretability, and many rely on single-modal data rather than multi-modal fusion, resulting in suboptimal performance for routine clinical use. Inherent biases in training data may also cause inaccurate predictions and exacerbate healthcare disparities in HF management. Third, translational application of AI in HF is constrained by practical barriers: advanced algorithms like reinforcement learning remain in basic research with limited clinical translation; unified evaluation criteria for AI models are lacking, hindering large-scale clinical promotion; and data security and ethical regulations restrict the accumulation of diverse clinical training data. Finally, current AI-related HF research is dominated by retrospective observational studies, with a shortage of prospective interventional trials to validate its clinical outcome benefits. Inadequate integration of clinical expertise and AI algorithm design also creates a gap between technological advances and real-world clinical practice in HF management.

## Conclusion

This review comprehensively presents the application of AI in a various links of HF management, from clinical diagnosis to continuous remote telecare in specific research scenarios. It also presents the current application limitations and challenges of AI, as well as its potential research value and application prospects in advancing clinical research and practice. Notably, it articulates the clinical aspiration of cardiologists to establish a standardized, process-oriented decision-making system for HF assisted by AI, which is designed to generate actionable, evidence-based recommendations for clinicians in specific clinical scenarios and liberate them from time-consuming, repetitive routine work through in-depth research, multi-center validation and model optimization in the future.

## Supplementary Information

Below is the link to the electronic supplementary material.

Supplementary Material 1.

## Data Availability

No datasets were generated or analysed during the current study.

## Abbreviations

HF: Heart failure
CVD: Cardiovascular disease
HFpEF: HF with preserved ejection fraction
AI: Artificial intelligence
ML: Machine learning
CRT: Cardiac resynchronization therapy
HFmrEF: HF with mid-range ejection fraction
HFrEF: HF with reduced ejection fraction
DL: Deep learning
ECG: Electrocardiogram
DL-CNN: Deep learning-based convolutional neural network
CNN-LSTM: Convolutional neural network-long short-term memory
CHF: Congestive heart failure
OS: Oxidative stress
RF: Random forest
PCP-HF: Pooled cohort equations to prevent HF
EHR: Electronic health records
HT: Heart transplantation
AF: Atrial fibrillation
CRT-D: Cardiac resynchronization therapy defibrillators
ICD: Implantable cardioverters-defibrillators
PASSION-HF: Patient Self-care uSing eHealth In chrONic Heart Failure
HFRS: Heart failure risk status
