# Supplementary material for: Artificial intelligence in heart failure
Source: Egypt Heart J. 2026 Mar 1;78:14. doi: 10.1186/s43044-026-00723-w (PMC12950836; doi:10.1186/s43044-026-00723-w)
Supplement: Supplementary file 1 — Supplementary Material 1. [file 43044_2026_723_MOESM1_ESM.docx]

**Table 1 subgroup analysis of AI in HF**

| Author (Ref.) | Learning types | Algorithm | Number of cases | Definitions of phenogroups and groups | Study Limitation |
| --- | --- | --- | --- | --- | --- |
| Shah et al. [42] | Unsupervised& supervised | Unbiased hierarchical cluster analysis, penalized model-based clustering, SVM | 397 patients | *Phenogroup 1*: the least electric and myocardial remodeling and dysfunction and the least hemodynamic derangement; *Phenogroup 2*: the worst LV relaxation, highest PCWP, and highest pulmonary vascular resistance; *Phenogroup 3*: the most severe electric and myocardial remodeling with the longest QRS duration, largest QRS-T angle, highest relative wall thickness and LV mass index, highest E/e′ ratio, and worst RV function | Single-center study, with validation only in an independent cohort from the same center, lacking external validation across multiple centers. |
| Kao et al.[43] | Unsupervised | LCA | 4113 patients | *Subgroup A*: men, alcohol use, low rates of AF, renal dysfunction, and valvular disease; *Subgroup B*: women more than men, anemia, low rates of AF, renal dysfunction, and valvular disease; *Subgroup C*: high rates of obesity, DM, hyper-lipidaemia, CAD, and anemia with worse renal function; *Subgroup D*: women with average rates of DM, hyper-lipidaemia, obesity, and renal insufficiency; *Subgroup E*: men with lower BMI, excess AF, and CAD; *Subgroup F*: older women with lower BMI and high rates of AF, valvular disease, renal dysfunction, and anemia | Based solely on demographic and clinical history data, without incorporating multidimensional data such as imaging or biomarkers |
| Przewlocka-Kosmala et al.[44] | Unsupervised | Automated hierarchical clustering | 228 (177 HFpEF and 51 normal) | *Subgroup 1*: deficient HR reserve;  *Subgroup 2*: not meeting the subgroup 1 criteria | Small sample size, with only internal validation performed, lacking external validation, and relying on a single dimension for classification. |
| Cohen et al. [45] | Unsupervised | LCA | 3445 patients | *Phenogroup 1*: younger, with mild symptoms;  *Phenogroup 2*: older, with stiff arteries, small LVs and AF;  *Phenogroup 3*: obese, diabetic, with advanced symptoms | Only internal stratified validation was conducted, with no independent external validation cohort, and no interventional studies were carried out. |
| Segar et al.[46] | Unsupervised | Penalized finite mixture model-based clustering analysis | 1767 HFpEF | *Phenogroup 1*: higher burden of co-morbidities, natriuretic peptides, and abnormalities in LV structure and function;  *phenogroup 2*: lower prevalence of cardiovascular and non-cardiac co-morbidities but higher burden of diastolic dysfunction;  *phenogroup 3*: lower natriuretic peptide levels, intermediate co-morbidity burden, and the most favorable diastolic function profile | Multi-omics data were not integrated, and the classification lacks strong association with long-term clinical outcomes. |
| Hedman et al.[47] | Unsupervised& supervised | Clustering, Elastic Net, Neural Networks and Naive Bayes | 539 HFpEF | *Phenogroup 1*: Younger, risk factors such as hypertension, CAD and diabetes, larger left ventricular volumes, and CKD; *phenogroup 2*: Older, less hypertrophy but worse LV and RV systolic function and more severe HF, and notably high prevalence of COPD; *phenogroup 3*: Younger, male, obesity, less cardiac diastolic and systolic function, and milder HF; *phenogroup 4*: Male, hypertension, LV hypertrophy, LA enlargement and AF; *phenogroup 5*: older, female, hypertension, CAD, large left side but good RV function; *phenogroups 6*: Older, female, low BMI, hypertension, AF and right-sided failure | Only internal validation was performed, with an excessive number of subtypes, and clinical utility remains to be verified. |
| Sotomi et al.[48] | Unsupervised | LCA | 1095 patients | *Group 1*: rhythm trouble, *group 2*: ventricular-arterial uncoupling, *group 3*: low output and systemic congestion, *group 4*: systemic failure | Based solely on laboratory and ultrasound data, validated only internally within a single center, without external validation. |
| Pandey et al.[49] | Unsupervised deep learning | DeepNN, TDA | 1242 patients | *Low risk groups*：younger, fewer comorbidities, less abnormalities in cardiac structure and function  *High risk groups*：older, a higher burden of comorbidities, greater abnormalities in cardiac structure and function with higher E/e’ ratio, LV mass, EF, and LAVI | Classification based only on ultrasound parameters, without incorporating clinical or biomarker data. |
| Woolley et al.[50] | Unsupervised | Hierarchical clustering algorithm | 429 patients | *cluster 1*: the highest prevalence of DM and renal disease; *cluster 2*: oldest age and frequent age-related comorbidities; *cluster 3*: youngest age, largest body size, least symptoms and lowest N-terminal pro-B-type natriuretic peptide (NT-proBNP) levels; *cluster 4*: highest prevalence of ischemic etiology, smoking and chronic lung disease, most symptoms, as well as highest NT-proBNP and troponin levels | Classification based solely on biomarkers, validated only internally within a single center, with no interventional studies to verify its value in guiding treatment. |

*LCA* latent class analysis, *HR* heart rate, *deepNN* deep neural network, *TDA* topological data analysis, *SVM* support vector machine, *CAD* coronary artery disease, *CKD* chronic kidney disease, *COPD* chronic obstructive pulmonary disease, *LV* left ventricle, *LA* left atrium, *BNP* brain natriuretic peptide, *BMI* body mass index, *AF* atrial fibrillation, *DM* diabetes mellitus, *EF* ejection fraction, *LAVI* left atrial volume index, *PCWP* pulmonary capillary wedge pressure

**Table 2 the application of machine learning algorithms in risk prediction in HF**

| Author (Ref.) | Algorithm | Objective | Sample sizes | Data sources | Validation status | Primary outcomes | Results | Study Limitation |
| --- | --- | --- | --- | --- | --- | --- | --- | --- |
| Pandey et al.[49] | DeepNN, TDA | Predicting the risk of hospitalization and death in high-risk groups | 1242 patients | echocardiographic parameters | External validation | a composite of all-cause death and hospitalization related to HF | High-risk phenogroup showed higher rates of HF hospitalization or cardiac death (HR: 1.92; 95% CI: 1.16 to 3.22; p =0.01) and higher event-free survival with spironolactone therapy (HR: 0.65; 95% CI: 0.46 to 0.90; p =0.01) | Only based on ultrasound parameters, without incorporating clinical, biomarker, and other multidimensional data. |
| Mortazavi et al.[51] | RF, boosting, RF combined hierarchically with SVM or LR, and Poisson regression | Predicting readmissions | 977 patients | the clinical data and patients’ socioeconomic, psychosocial, and health status | Internal validation | all-cause readmission or mortality within 180 days | In 30-day all-cause readmission prediction, RF’s C statistics is 0.628. For readmissions because of HF, boosting’s C statistic is 0.678. In 180-day all-cause readmission prediction, the RF in SVM hierarchical method’s C statistic is 0.65 | Small sample size, with only internal validation, without accounting for regional and healthcare resource variations. |
| Awan et al.[52] | multi-layer perceptron, PCA | Determining the most relevant and also transform variables for the prediction of30-day readmission or death in HF | 10757 patients | Demographics, admission characteristics, medical history, socio-economics, medication history, out-of-hospital healthcare services, emergency inpatient admission | Internal validation | 30-day readmission or death | prediction model (AUC 0.62)  Transformation of the original 47 variables (AUC 0.66) | Only internal validation; limited performance improvement after variable transformation, and no external validation conducted. |
| Greenberg et al.[53] | MARKER-HF (Previous studies) | To predict mortality and distinguish varying levels of risk for patients | 4064 patients | Echocardiographic measurement | External validation was completed in the preliminary stage, and this study further verifies the efficacy | Mortality | C-statistics ranging between 0.83 to 0.89(Only based on ultrasonic indicators) | Only based on ultrasound indicators; external validation failed to reproduce the highest values, and clinical data were not included. |
| Sharma et al.[54] | XGBoost model | Predicting risk of 30-day readmission | 9845 patients | Administrative Data | External validation | unplanned all-cause hospital readmission within 30 days | the highest AUROCs with XGBoost being the highest at 0.65; the LACE score was at 0.57 | Only based on administrative data, with limited information dimensions and moderate predictive performance. |
| Polo et al.[55] | MICE, LASSO algorithm, XGBoost, Ada Boost Classifier, RF, Gradient Boosting | Predicting unplanned all-cause 30-day readmissions in HF elderly patients | 3079 patients | EHR: demographics, medical history, physical examinations, diagnoses, procedures, labs, and medications | Internal validation | All-cause readmissions within 30 days from the index hospitalization event | In the test cohort AUCs (IC95%) of XGBoost, Ada Boost Classifier, RF, and Gradient Boosting, and LACE Index were: 0.803, 0.782, 0.776, 0.786, and 0.504, respectively, for predicting readmissions | Only internal validation, focused on the elderly HF population, with no validation data from younger populations. |
| Heitzinger et al. [56] | RSF | To provide a streamlined approach to risk-stratification | 4868 patients (3359 moderate and 1509 severe s TR) | Clinical, echocardiographic and laboratory parameters | Internal validation | all-cause mortality | 4 or 5 adverse features had a fourfold risk increase in moderate sTR [4.81(3.56–6.50) HR 95%CI, P < 0.001] and fivefold risk increase in severe sTR [5.33 (3.28–8.66) HR 95%CI, P < 0.001] | Limited to HF patients with secondary tricuspid regurgitation, resulting in restricted applicability. |
| Adler et al.[58] | Decision tree algorithm | Predicting mortality risk among patients with HF | 14589 patients | The complete blood count, comprehensive metabolic panel, vital sign measurement, electrocardiogram, and echocardiogram | External validation | High-risk (died within 90 days of index event); low-risk (follow-up 800 (or more) days after index event and with no recorded date of death) | The risk score had AUC of 0.88, External validation in two separate HF populations gave AUCs of 0.84 and 0.81 | Model interpretability is moderate, lacking integration of novel data such as multi‑omics and wearable devices. |
| Wang et al.[59] | Feature rearrangement based deep learning | Proposing a fast and accurate HF mortality prediction framework | 10198 inpatients records | EHR | Internal validation | In-hospital mortality, 30-day mortality after discharge and 1-year mortality after discharge | The Chi2 rearrangement with Focal loss has the best performance in AACC (81.12%), Recall (80.55&) and AUC (0.888) | Only internal validation, based on single‑center electronic medical records, without cross‑center validation. |
| Wang et al.[60] | LR, KNN, SVM, NB, MLP, XGBoost | Prediction of 3-year all-cause mortality in patients with HF caused by CHD | 1562 patients | Demographics, medical history, physicals status and vitals, medical therapy, echocardiography, ECG and laboratory parameters | Internal validation | All-cause mortality throughout 3 years of follow up | Coronary heart disease induced HF population :Over the 3-year follow-up period, the XGBoost model achieved a mean AUC of 0.8207 (95% confidence interval [CI]: 0.8143–0.8272) and an F1-scoreof 0.4476 (95% CI: 0.4407–0.4546) for mortality | Only internal validation, focused on HF populations caused by coronary heart disease, limiting general applicability. |
| Segar et al.[61] | RF | Predicting in-hospital mortality in HF | 123634 patients | Recorded data | External validation | In-hospital mortality | C statistic 0.81 for Black patients and 0.82 for non-Black patients; External validation: C statistic 0.79 for Black patients and 0.80 for non-Black patients | Did not incorporate factors influencing mortality such as socioeconomic status and healthcare accessibility. |
| Segar et al.[62] | RF, oRSF, Forward-Cox, Ridge-Cox, Boosted-Cox, GBT, oRSF Top-20 | Predicting 10-year risk of HF | ARIC, 15792 participants; DHS,3557 participants; MESA,6814 participants; JHS,5306 participants | The ARIC (Atherosclerosis Risk in Communities Study), DHS (Dallas Heart Study), JHS (Jackson Heart Study), and MESA (Multi-Ethnic Study of Atherosclerosis) data sets | External validation | Incident HF | Black (JHS, C-index=0.88)，White (ARIC, C-index=0.89); External validation: Black (C-index=0.80–0.83)，White (C-index=0.82) | Based on data from European and American populations, lacking validation in Asian, African, and other populations. |
| Segar et al.[63] | stepwise backward selection, stepwise forward selection, RSF | predicting the risk of HF among patients with T2DM | 8756 patients | Demographics, clinical variables, laboratory data, electrocardiography, baseline antihyperglycemic therapies, and treatment randomization | External validation | Incident hospitalization or death due to HF | RSF models had the best discrimination (C-index= 0.77); External validation: C-index was0.74 for RSF models | Focused on type 2 diabetic populations, without including data on the severity of diabetes complications. |
| Gao[64] | feature-fusing DL network | predict the in-hospital outcomes for HF | 21814patients | 3 open-access clinical databases | External validation | predict the in-hospital outcome：survivor or nonsurvivor | AUROC 0.838 (95% CI 0.827-0.851); Prospective validation: 0.849 (95% CI 0.841-0.856); External validation: 0.767 (95% CI 0.762-0.772) | Based on publicly available databases, with data differing from real‑world clinical scenarios. |

*PCA* principal component analysis, *MICE* multivariate imputation via chained equations, *LASSO* least absolute shrinkage and selection operator, *HER* electronic health records, *MLP* multi-layer perceptron, *NB* naive Bayesian, *KNN* k-nearest neighbors, *LR* logistic regression, *SVM* support vector machines, *HT* heart transplantation, *SLR* stepwise logistic regression, *GB* gradient boosting, *CART* classification and regression tree, *GBM* gradient- boosted model, *EMR* electronic medical record, *AUPRCs* area under precision-recall curves, *ANN* artificial neural network, *AACC* average accuracy, *RSF* random survival forest

**Table** **3** the applications of ML in assessing the prognosis of pharmacotherapy, evaluating patients before and after receiving CRT and evaluating the prognosis of HT

| Author (Ref.) | | Algorithm | Objective | Sample sizes | Data sources | Validation status | Primary endpoint | Study limitation |
| --- | --- | --- | --- | --- | --- | --- | --- | --- |
| Karwath et al.[69] | | Neural network-based variational autoencoders and hierarchical clustering | To define clusters of β-blocker efficacy in patients with sinus rhythm and AF | 15659 patients | From 9 double-blind, randomized, placebo-controlled trials of β blockers | External validation | All-cause mortality | Limited to beta-blockers, without coverage of other HF therapeutic drugs. |
| Sotomi et al.[71] | | ML-based clustering model | To assess effectiveness of medications on clinical outcomes of the different phenotypes | 1100 patients | Basic patient characteristics, echocardiography, laboratory tests and lists of medications | Internal validation | A composite of all-cause death and HF hospitalization | Only internal validation; the classification was not analyzed in association with other medications. |
| Feeny et al.[77] | | Naive Bayes classifier | To predict CRT response compared with current guidelines | 925 patients | Clinical variable sets | Internal validation | Death, heart transplant, or placement of left ventricular assist device | Only internal validation; limited clinical variable dimensions without inclusion of key imaging indicators. |
| Cikes et al.[78] | | Multiple Kernel Learning and K-means clustering | To identify responders to CRT | 1106 patients | Clinical and echocardiographic characteristics | Internal validation | Death from any cause or a non-fatal HF event | Only internal validation, without integration of postoperative follow-up data for model optimization. |
| Howell et al.[79] | | RF, CNN, LASSO, adaptive LASSO, plugin LASSO, elastic net, ridge, LR | To identify CRT candidates | 741 patients (from the SMART-AV) | baseline clinical characteristics data, ECG and echocardiography, biomarkers | Internal validation | a composite of freedom from death and HF hospitalization and a >15% reduction in LV end systolic volume index at 6-month follow-up | Based solely on the SMART‑AV study cohort, with limited population applicability and no external validation. |
| Svyatoslav et al.[80] | | classification algorithms: LR, LDA, SVM with linear kernel, RF;  feature selection: RFMDA, UST, L1-based feature selection | to develop a predictive model of CRT outcome using clinical data recorded in patients before CRT | 57 patients | clinical data recorded in patients before CRT, model-derived biomarkers of ventricular excitation in the left bundle branch block mode of activation and under BiV stimulation | Internal validation | More than 10% increase in LV EF | The best ML classifier: AUC 0.82, Very small sample size, single‑center study, no external validation, and poor model generalizability. |
| Galli et al.[81] | | A combination of the Boruta algorithm and RF methods, the k-medoid method | To identify CRT candidates | 193 patients | Clinical, electrocardiographic, and echocardiographic data | Internal validation | LV end-systolic volume of ≥15% | Small sample size, only internal validation, without incorporation of biomarker data. |
| Wouters et al.[82] | | variational auto-encoder | To predict CRT outcome | 251473 patients (1.1 million ECGs) | ECG | Internal validation | Death, left ventricular assist device, or HT | Based only on ECG data, without inclusion of clinical or key imaging indicators, resulting in moderate performance. |
| Boehmer et al. [85] | | Multisensory-based algorithm | To predict HF events in patients with implanted devices | 900 patients | SENSOR DATA COLLECTION | Internal validation | Sensitivity to detect HFE >40% and unexplained alert rate <2 alerts per patient-year | Based solely on implanted device sensor data, with limited population applicability and no external validation. |
| Kalscheur et al.[86] | | Naive Bayes classifier, sequential minimal optimization for training a SVM, decision lists, J48 DT, and RF | To develop a model to predict outcomes after CRT | 595 patients with CRT-defibrillator | the COMPANION trial | Internal validation | All-cause mortality or HF hospitalization at 12 months post-CRT | Based only on the COMPANION trial cohort, without validation using real‑world clinical data. |
| Tokodi et al.[87] | | LR, ridge regression, SVM, KNN, gradient boosting classifier, RF, conditional inference RF, and multi-layer perceptron | To develop a machine learning (ML)-based risk stratification system to predict 1-, 2-, 3-, 4-, and 5year all-cause mortality from pre-implant parameters | 2282 patients | Pre-implant clinical characteristics | Internal validation | All-cause mortality | Only internal validation, without inclusion of factors affecting mortality such as postoperative follow-up and complications. |
| Puyol-Antón et al.[88] | | nnU-Net;  multimodal DL model: SVM classifier | To predict CRT response | 700 healthy subjects (only CMR), 10030 patients (only echocardiography), 50 HF and 50 CRT patients (CMR+ echocardiography), 12 CRT patients (only echocardiography) | UK Biobank; EchoNet-Dynamic; Guys and St Thomas NHS Foundation Trust; GSTFT CRT echocardiography database | Internal validation | - | Imbalanced sample size ratio, only internal validation, and limited integration of multimodal data. |
| Feeny et al.[89] | | Principal component analysis, k-means clustering | To identify CRT patients with differential outcomes | 946 CRT patients with conduction delay | ECG | Internal validation | (1) composite end point of death, left ventricular assist device, or heart transplant, and (2) degree of echocardiographic left ventricular ejection fraction (LVEF) change after CRT | Based only on ECG data, without inclusion of clinical or imaging data, resulting in limited applicability. |
| Gupta et al.[90] | | SLR, GB, RF | To develop a model for predicting prolonged hospital length of stay after pediatric HT | 4414 patients undergoing HT | Demographics and other patient data, procedural data at HT, and event data, transplantation, follow up and death | Internal validation | at the time of HT with a recorded discharge date | Limited to pediatric HT patients, without inclusion of factors such as postoperative complications and nutritional status. |
| Desai et al.[91] | | LASSO, CART, RF, GBM | To predict key outcomes in patients with HF and evaluating the added value of augmenting claims based predictive models with electronic medical record (EMR)–derived information | 9502 patients | Medicare claims data, EMRs | External validation | All-cause mortality, HF hospitalization, total costs for all-causes, the number of days patients spent at home | Low AUPRC values, poor cost prediction performance, and absence of patient‑reported subjective factors. |
| Zhou et al.[92] | | LR, RF, GBM, SVM, XGBoost, AdaBoost, ANN | To Predict 1-year mortality after HT | 381 patients | EMR and outpatient review and telephone | Internal validation | 1-year mortality | Single‑center study with small sample size, without inclusion of core factors such as donor data and immune rejection. |
| Agasthi et al.[93] | | GBM | To predict survival and graft failure (GF) 5 years after OHT | 78852 patients | data on almost 400 variables before, during, and after transplantation until death | Internal validation | All-cause mortality and graft failure at 5 years after OHT | Only internal validation; excessive variable dimensions leading to poor model interpretability and lack of feature selection. |
|  |  |  |  |  |  |  |  |  |
| Medved et al.[94] | | DL | To compare the accuracy of two risk models (IHTSA & IMPACT) to predict survival after HT | 27705 patients | Includes almost 500 variables that encompass recipient, donor, and transplant information | Internal validation | 1-year mortality | Only compared two existing models without constructing a new model or incorporating postoperative follow-up data. |
| Kampaktsis et al.[95] | | Adaboost, SVM, K-nearest neighbor, DT, LR | To increase the predictive accuracy of mortality after HT | 18625 patients | baseline data and follow up survival data | Internal validation | 1, 3, 5-year mortality | Only internal validation; significant decline in long‑term predictive performance, without inclusion of immunosuppressive therapy data. |
| Ayers et al.[96] | | DNN, LR, AdaBoost and RF | To improve prediction of survival after OHT | 33657 patients (525 variables) | the United Network for Organ Sharing data | Internal validation | 1‐year post‐transplant survival | Based solely on the UNOS database, without real‑world clinical scenario validation and with excessive variable dimensions. |

*AF* atrial fibrillation, *EHR* electronic health record, *HR* hazard ratio, *CI* confidence interval, *CRT* cardiac resynchronization therapy, *SMART-AV* SmartDelay-Determined AV Optimization: Comparison of AV Optimization Methods Used in CRT, *ECG* electrocardiography, *LDA* linear discriminant analysis, *RFMDA* random forest mean decrease accuracy, *UST* univariate statistical testing, *TAPSE* Tricuspid annular plane systolic excursion, *HFEs* HF events, *CMR* cardiac magnetic resonance, *EMR* electronic medical records, *OHT* orthotopic heart transplant, *GF* graft failure, *IHTSA* International Heart Transplantation Survival Algorithm, *DNN* deep neural network, *RF* random forest
